# Umbilical Cord Blood Glucose Concentrations and Transitional Neonatal Hypoglycemia

**DOI:** 10.1001/jamanetworkopen.2026.6170

**Published:** 2026-04-20

**Authors:** Marcia Roeper, Thomas Meissner, Lisa Friesl, Calvin Kurz, Mark Dzietko, Ertan Mayatepek, Henrike Hoermann, Sebastian Kummer

**Affiliations:** 1 Department of General Pediatrics, Neonatology and Pediatric Cardiology, Medical Faculty, University Hospital Düsseldorf, Heinrich-Heine-University, Düsseldorf, Germany

## Abstract

**Importance:**

Neonatal hypoglycemia is a common metabolic disorder that may cause long-term neurodevelopmental impairment. Reliable early biomarkers to estimate hypoglycemia risk are lacking.

**Objective:**

To evaluate whether umbilical cord blood glucose (UCBG) values and derived parameters are associated with transitional neonatal hypoglycemia (TNH) and to establish percentile-based UCBG reference values.

**Design, Setting, and Participants:**

This prospective cohort study was performed at a tertiary perinatal center in Düsseldorf, Germany. Data were collected from May 27, 2020, to September 1, 2022. The final dataset included neonates (gestational age, ≥35 weeks 0 days) with paired arterial and venous umbilical cord blood samples who were exposed or unexposed to hypoglycemia risk factors. Exclusion criteria consisted of incomplete or invalid cord blood samples, missing glucose measurements within the first 4 hours of life, and transient or persistent hyperinsulinism. Maternal and neonatal clinical data were systematically recorded. Data were analyzed from March 18 to July 15, 2025.

**Exposures:**

Paired arterial and venous cord blood samples collected at birth; standardized postnatal glucose screening and management.

**Main Outcomes and Measures:**

The primary end point was hypoglycemia, defined as a glucose level of 45 mg/dL or less during postnatal screening; severe hypoglycemia was defined as a glucose level of less than 30 mg/dL. UCBG parameters included arterial and venous glucose values, venous-arterial difference, and glucose extraction rate. Prospective association of UCBG parameters with postnatal hypoglycemia was assessed using receiver operating characteristic (ROC) analyses.

**Results:**

Of 598 neonates included in the analysis, the median gestational age was 39 weeks 0 days (IQR, 37 weeks 6 days to 40 weeks 0 days), and 332 (55.5%) were male. Hypoglycemia occurred in 188 neonates (31.4%) and severe hypoglycemia occurred in 22 neonates (3.7%). The median arterial UCBG level was 67 (IQR, 58-84) mg/dL; venous UCBG level, 85 (IQR, 72-102) mg/dL; venous-arterial level difference, 16 (IQR, 10-23) mg/dL; and extraction rate, 19.0% (IQR, 12.2%-25.5%). Arterial UCBG values of 45 mg/dL or less occurred in 22 neonates (3.7%). Compared with neonates with levels greater than 45 mg/dL, those with levels of 45 mg/dL or less had significantly higher median venous-arterial UCBG level differences (15 [IQR, 10-23] mg/dL vs 24 [IQR, 17-38] mg/dL), UCBG extraction rates (18.7% [IQR, 11.9%-25.0%] vs 35.3% [IQR, 27.5%-56.1%]), and higher incidence of severe TNH (3.3% [19/576] vs 13.6% [3/22]). ROC analyses demonstrated poor discriminative performance for all UCBG parameters (maximum area under the ROC curve, 0.64 [95% CI, 0.45-0.81]), and no percentile-based cutoff achieved clinically useful sensitivity or specificity. Percentiles for UCBG values are provided.

**Conclusions and Relevance:**

The findings of this prospective cohort study suggest that UCBG parameters were not associated with subsequent TNH. However, this study provides a large, single-center, percentile-based UCBG dataset that can support future research and clinical interpretation. Very low UCBG values may help identify neonates with higher likelihood of pathologic hypoglycemia, but this requires further validation.

## Key Points

**Question:**

Are umbilical cord blood glucose (UCBG) values and derived parameters associated with transitional neonatal hypoglycemia (TNH)?

**Findings:**

In this cohort study of 598 neonates, 31% developed hypoglycemia at 45 mg/dL or less and 4% at less than 30 mg/dL. Arterial and venous UCBG values, venous-arterial difference, and extraction rates were not associated with subsequent TNH.

**Meaning:**

These findings suggest that UCBG parameters were not associated with subsequent TNH; however, this study provides a large, percentile-based UCBG dataset to guide future research and interpretation of neonatal glycemia.

## Teaser

This cohort study evaluates the association of umbilical cord blood glucose values and derived parameters with transitional neonatal hypoglycemia in neonates.

## Introduction

Transitional neonatal hypoglycemia (TNH) is the most common metabolic disorder in neonates and affects as many as 15%^1^ of all newborns and 50% of those with specific risk factors (eg, maternal diabetes, prematurity, being born large or small for gestational age, and perinatal stress).^2^ After cessation of transplacental glucose delivery at birth, neonatal plasma glucose concentrations typically decline to a nadir within approximately 2 hours and subsequently rise gradually to adult-equivalent levels by approximately the fourth day of life.^3,4^ Thus, TNH reflects a physiologic adaptation process from fetal to extrauterine life.^4^ Although typically brief, it may result in glucose concentrations below common treatment thresholds, especially in at-risk newborns^3^ who may have increased glucose consumption, limited glycogen storages, or increased insulin secretion.^5^

As glucose is the primary energy source for the developing brain, severe or prolonged hypoglycemia may result in adverse neurodevelopment.^6,7^ Because clinical signs lack both specificity and sensitivity,^8^ it is common practice to perform glucose screening in neonates at risk. However, screening practices and guideline recommendations vary widely,^9^ often missing individuals without identifiable risk factors. This includes infants with severe hypoglycemic disorders such as congenital hyperinsulinism, who experience high rates of neurologic impairment.^7,10,11,12,13^ Consequently, there is growing interest in identifying early markers that may estimate the risk of postnatal hypoglycemia, ideally at birth.^14,15^

Umbilical cord blood glucose (UCBG) values indicate the child’s metabolic state around the time of delivery. It has been hypothesized that arterial UCBG levels and derived parameters such as venous-arterial difference and glucose extraction rate may offer insight into fetal glucose consumption and postnatal hypoglycemia risk. However, only 2 studies^16,17^ have been published, both conducted in selective cohorts and focused on very specific aspects of UCBG metabolism and postnatal glycemia. Neither study provides normative reference data, and methodologic limitations are evident in both, particularly in the absence of validated protocols for arterial vs venous sample differentiation.^16,17^

This prospective cohort study evaluated UCBG levels in neonates with and without TNH risk factors, correlated them with postnatal glycemia, and assessed their utility as an indicator of postnatal TNH in clinical practice. The study also provides percentile-based reference data on UCBG values and glucose extraction rates from a large, well-characterized cohort of term and late preterm neonates at a tertiary care birth center.

## Methods

### Study Design and Population

This cohort study was approved by the Ethics Committee of the Medical Faculty of the Heinrich-Heine-University Düsseldorf and was conducted in accordance with the Declaration of Helsinki.^18^ Written informed consent was obtained from both parents of each participating neonate. We followed the Strengthening the Reporting of Observational Studies in Epidemiology (STROBE) reporting guideline.

Data were derived from 1018 neonates (gestational age, ≥35 weeks 0 days) enrolled in 3 prospective cohort studies on TNH at the University Children’s Hospital Düsseldorf, Germany, between May 27, 2020, and September 1, 2022.^8,19,20^ Of the total cohort, 857 neonates had predefined risk factors for TNH (exposed group), and 161 had no risk factors (unexposed group). Risk factors included maternal diabetes, late preterm birth (gestational age, 35 weeks 0 days to 36 weeks 6 days), large or small for gestational age (birth weight <10th or >90th percentile), fetal growth restriction, perinatal stress (including asphyxia, respiratory distress, arterial umbilical cord blood pH <7.1, vacuum extraction, or unplanned cesarean delivery for pathologic cardiotocography), and hypothermia. Exposed neonates underwent standardized prevention, blood glucose screening, and management per institutional TNH protocol.^19^

### UCB Sampling and Validation and Exclusion Criteria

Immediately after cord clamping, paired blood samples were collected from the arterial and venous umbilical cord vessels and analyzed promptly using a calibrated blood gas analyzer (ABL800 FLEX; Radiometer). Glucose levels were measured in a range of 0.0 to 1080 mg/dL (to convert to mmol/L, multiply by 0.0555). To validate venous and arterial assignment, only pairs meeting predefined criteria were included: pH difference at least 0.02, arteriovenous Pco_2_ difference 3.8 mm Hg or greater, and a positive venous-arterial difference in oxygen saturation.^21,22^

Incomplete pairs and those not meeting validation criteria were excluded, as were neonates with missing blood glucose values within the first 4 hours of life (Figure). Three neonates with diagnoses of transient or persistent hyperinsulinism were excluded due to a different pathomechanism leading to hypoglycemia. Sensitivity analysis revealed no significant differences between included and excluded neonates beyond those attributable to exclusion criteria, except higher vaginal delivery rates in excluded neonates (eTable 1 in Supplement 1). No participants were lost to follow-up, as all measurements occurred during initial hospitalization.

**Figure.**
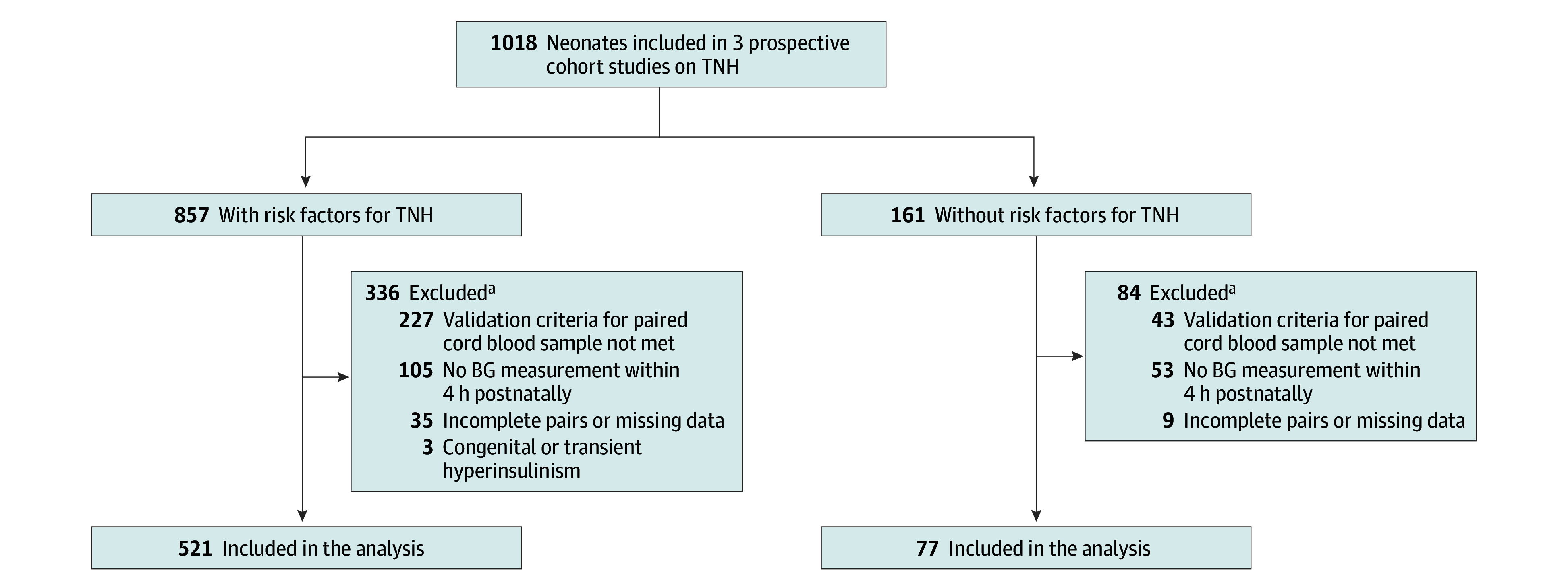
Study Flow Diagram BG indicates blood glucose; TNH, transitional neonatal hypoglycemia. ^a^Some neonates met more than 1 exclusion criterion.

Maternal and neonatal clinical data were extracted from medical charts. Data were systematically recorded in a study database using Claris FileMaker Pro, version 19 (Claris International Inc).

### Postnatal Glucose Monitoring

Capillary blood glucose level was measured using a glucose oxidase–based method (StatStrip Glucose Meter; Nova Biomedical) with a measuring range of 10 to 600 mg/dL and coefficient of variation less than 5% across the entire analytical measurement range.^23^ Blood gas analyzers (ABL800 FLEX or ABL90 FLEX PLUS; Radiometer) were used when additional blood gas parameters were required (<5% of samples); for the purposes of this study, blood glucose refers to the plasma-calibrated glucose result from both devices. In exposed neonates, the first glucose measurement was obtained before the second feed, approximately 2 to 3 hours after birth, with subsequent measurements per clinical protocol. Unexposed neonates underwent 2 scheduled measurements at 2 to 3 hours of life and between 36 to 72 hours at the time of routine newborn screening. If hypoglycemia was detected, the same management protocol as for exposed neonates was applied.^19^

### Hypoglycemia Prevention

Neonates with TNH risk factors received standardized preventive measures beginning within 30 minutes postnatally: uninterrupted skin-to-skin contact, thermal stability, and early breastfeeding initiation. If breastfeeding was not feasible, formula (10-20 mL) or oral dextrose gel was administered.^19^

### Statistical Analysis

Data were analyzed from March 18 to July 15, 2025, using SPSS Statistics, version 29.0 (IBM Corp). Continuous variables are summarized as medians with IQRs; categorical variables, as counts and percentages. Group differences are reported with 95% CIs as inferential measures of precision.

Normality of continuous variables was assessed using the Shapiro-Wilk test and visual inspection of distributions. As most variables were nonnormally distributed and transformation would compromise clinical interpretability, nonparametric methods were used.

Between-group comparisons of continuous variables were performed using the Mann-Whitney test (2 groups) or Kruskal-Wallis test (>2 groups). Categorical variables were compared using the χ^2^ test or Fisher exact test. Associations between continuous variables were assessed using Spearman rank correlation coefficients (ρ values).

Discriminative performance of UCBG parameters for TNH was assessed using receiver operating characteristic curves (ROCs) with the area under the curve (AUROC) and corresponding 95% CIs, sensitivity, specificity, and positive and negative predictive values. Multivariable logistic regression was performed to assess association of UCBG parameters and TNH adjusted for covariates. Statistical significance was defined as a 2-sided *P* < .05. Given the exploratory nature of analyses, no adjustment for multiple comparisons were performed. Analyses used available data without imputation.

Post hoc sensitivity analysis for the primary end point (TNH ≤45 mg/dL) demonstrated greater than 95% power to detect an AUROC of 0.600 or greater (SE, 0.025; equivalent Cohen *d*, 0.56) compared with the null hypothesis of AUROC = 0.50. The UCBG extraction was calculated according to Kennedy et al^16^ as [(venous UCBG – arterial UCBG)/venous UCBG] × 100. Voigt percentiles were calculated for birth measures.^24^ TNH was defined as a blood glucose level of 45 mg/dL or less; severe TNH, as a blood glucose level of less than 30 mg/dL.

## Results

Of 1018 neonates, 598 (58.7%) were included in the analyses; 266 (44.5%) were female and 332 (55.5%) were male; and the median gestational age was 39 weeks 0 days (IQR, 37 weeks 6 days to 40 weeks 0 days). Of these, 521 neonates (87.1%) were exposed and 77 (12.9%) were unexposed to hypoglycemic risk factors.

### Descriptive Statistics of Postnatal Glycemia

During postnatal glucose screening, 188 neonates (31.4%) experienced at least 1 episode of TNH and 22 (3.7%) experienced at least 1 episode of severe TNH. Overall, 85 neonates (14.2%) had TNH and 17 (2.8%) had severe TNH at the initial measurement within the first 4 hours of life (Table 1). Only 1 unexposed neonate had severe TNH; however, 15 of 77 unexposed neonates (19.5%) experienced at least 1 episode of TNH.

**Table 1.** Descriptive Characteristics of the Study Cohort^a^

Characteristic	Study group		*P* value
	Neonates exposed to hypoglycemia risk factors (n = 521)	Neonates unexposed to hypoglycemia risk factors (n = 77)	
Gestational age, median (IQR), wk + d	39 + 0 (37 + 5 to 40 + 0)	39 + 0 (38 + 3 to 39 + 6)	.26
Sex			
Male	292 (56.0)	40 (51.9)	.50
Female	229 (44.0)	37 (48.1)	.50
Singleton pregnancy	457 (87.7)	73 (95.8)	.67
Birth weight, median (IQR), g	3210 (2700-3830)	3460 (3238-3873)	.002
Birth weight SDS, median (IQR)	−0.2 (−1.3 to 1.0)	0.1 (−0.3 to 0.9)	.01
Vaginal delivery	196 (37.6)	20 (26.0)	.047
Cesarean delivery	325 (62.4)	57 (74.0)	.047
Elective	168 (32.2)	47 (61.0)	<.001
Unplanned	157 (30.1)	10 (13)	<.001
Apgar score, median (range)			
1 min	9 (1-10)	9 (8-9)	.008
5 min	10 (3-10)	10 (8-10)	<.001
10 min	10 (5-10)	10 (9-10)	.009
Maternal IV glucose infusion during delivery	27 (5.2)	5 (6.5)	.59
No. of risk factors for TNH, median (IQR)	2 (1-2)	NA	NA
Risk factor for TNH^b^			
Maternal diabetes in pregnancy	228 (43.8)	NA	NA
Small for gestational age^c^ and/or fetal growth restriction	144 (27.6)	NA	NA
Large for gestational age^d^	108 (20.7)	NA	NA
Late preterm birth^e^	103 (19.8)	NA	NA
Perinatal stress	123 (23.6)	NA	NA
Hypothermia	137 (26.3)	NA	NA
No. of BG measurements, median (IQR)	7 (4-12)	3 (2-5)	<.001
Age at first BG measurement, median (IQR), min	158 (133-178)	152 (133-169)	.36
Lowest BG value, median (IQR), mg/dL	50 (43-58)	55 (49-65)	<.001
Age at lowest BG value, median (IQR), min	401 (189-861)	214 (149-2368)	.14
First BG level ≤45 mg/dL	75 (14.4)	10 (13)	.74
First BG level <30 mg/dL	17 (3.3)	0	.15
≥1 BG level ≤45 mg/dL	173 (33.2)	15 (19.5)	.02
≥1 BG level <30 mg/dL	21 (4.0)	1 (1.3)	.34
Arterial UCBG ≤45 mg/dL	20 (3.8)	2 (.03)	>.99
Arterial UCBG <30 mg/dL	3 (0.6)	0	>.99

Abbreviations: BG, blood glucose; IV, intravenous; NA, not applicable; SDS, standard deviation score; TNH, transitional neonatal hypoglycemia; UCBG, umbilical cord blood glucose.

SI conversion factor: To convert blood glucose to mmol/L, multiply by 0.0555.

^a^ Data are presented as the No. (%) of neonates unless indicated otherwise.

^b^ Infants could have more than 1 risk factor.

^c^ Defined as birth weight less than the 10th percentile.

^d^ Defined as birth weight greater than the 90th percentile.

^e^ Defined as gestational age 35 weeks 0 days to 36 weeks 6 days.

### Descriptive Statistics of UCBG Parameters

The median arterial UCBG level was 67 (IQR, 58-84) mg/dL; the median venous UCBG level was 85 (IQR, 72-102) mg/dL. The median venous-arterial level difference was 16 (IQR, 10-23) mg/dL; the median glucose extraction rate was 19.0% (IQR, 12.2%-25.5%). Percentiles of umbilical cord blood measurements are provided in Table 2; percentiles for exposed vs unexposed neonates and overall are provided in eTable 2 in Supplement 1 and percentiles differentiated by risk factor and compared with unexposed neonates are provided in eTable 3 in Supplement 1. Arterial UCBG and the first blood glucose levels showed a weak correlation (ρ = 0.102 [*P* = .01]). The venous-arterial difference and the glucose extraction rate did not correlate with the first postnatal blood glucose value (ρ = 0.053 [*P* = .20] and ρ = 0.014 [*P* = .75], respectively). Compared with unexposed neonates, exposed neonates had significantly higher venous (76 [IQR, 70-88] mg/dL vs 86 [IQR, 73-104] mg/dL [*P* < .001]) and arterial (60 [IQR, 56-74] mg/dL vs 68 [IQR, 58-85] mg/dL [*P* = .001]) UCBG levels (Table 2). The initial blood glucose levels were nearly identical between groups (63 [IQR, 52-72] mg/dL for exposed neonates vs 62 [IQR, 53-72] mg/dL for unexposed neonates [*P* = .95]), yet exposed neonates had a greater decline in glucose level from birth to first postnatal measurement than unexposed neonates (7 [IQR, −7 to 26] mg/dL vs 3 [IQR, −22 to 14] mg/dL [*P* = .02]). Venous-arterial gradients or extraction rates did not differ between groups.

**Table 2.** Cord Blood Parameters and Glycemia in Neonates^a^

Parameter	Study group					*P* value^b^
	Neonates exposed to hypoglycemia risk factors (n = 521)		Neonates unexposed to hypoglycemia risk factors (n = 77)			
	Median (IQR)	5th to 95th percentile		Median (IQR)	5th to 95th percentile	
Arterial pH	7.28 (7.23-7.31)	7.14-7.34		7.29 (7.25-7.32)	7.16-7.35	.08
Venous pH	7.35 (7.32-7.38)	7.25-7.42		7.37 (7.34-7.39)	7.27-7.43	.006
Arterial base excess level, mEq/L	−3.5 (−6.1 to −1.8)	−10.3 to 0.1		−2.2 (−4.2 to −1.0)	−9.4 to 1.5	<.001
Arterial Pco_2_, mm Hg	52.4 (48.1-57.8)	41.4-67.9		52.0 (47.5-56.8)	42.8-64.4	.63
Venous Pco_2_, mm Hg	41.2 (37.4-44.8)	30.1-51.6		40.8 (36.4-43.2)	30.5-51.3	.33
Arterial lactate level, mg/dL	32.4 (22.5-46.8)	15.3-71.2		26.1 (20.7-36.0)	14.4-66.7	.02
Arterial UCBG level, mg/dL	68 (58-85)	47-118		60 (56-74)	46-97	.001
Venous UCBG level, mg/dL	86 (73-104)	62-132		76 (70-88)	61-118	<.001
First BG level, mg/dL	63 (52-72)	34-91		62 (53-72)	38-88	.95
Venous-arterial UCBG level difference, mg/dL	16 (10-24)	−4 to 41		16 (10-21)	3-32	.55
Arterial UCBG level and first BG level difference, mg/dL	7 (−7 to 26)	−24 to 63		3 (−22 to 14)	−27 to 44	.02
UCBG extraction rate, %^c^	18.8 (12.0-25.6)	−4.3 to 39.1		21.1 (14.1-24.9)	2.9-33.0	.46

Abbreviations: BG, blood glucose; UCBG, umbilical cord blood glucose.

SI conversion factors: To convert arterial base excess to mmol/L, multiply by 1; blood glucose to mmol/L, multiply by 0.0555; lactate to mmol/L, multiply by 0.111.

^a^ eTable 1 in Supplement 1 provides a separate analysis of different exposed subgroups and unexposed neonates.

^b^ Calculated using the Mann-Whitney test.

^c^ Calculated as [(venous UCBG – arterial UCBG)/venous UCBG] × 100.

Neonates born vaginally had higher arterial and venous UCBG concentrations, as well as more pronounced biochemical indicators of birth stress, including lower pH, reduced base excess levels, and higher lactate levels (eTable 4 in Supplement 1). Compared with cesarean deliveries, vaginal deliveries were associated with higher venous-arterial UCBG level difference (14 [IQR, 10-19] mg/dL vs 22 [IQR, 11-32] mg/dL [*P* < .001]) and glucose extraction rate (17.9% [IQR, 12.7%-23.8%] vs 22.9% [IQR, 9.1%-30.4%] [*P* = .004]).

Despite these physiologic differences, TNH incidence did not differ by delivery mode in the first blood glucose measurement (TNH: 30 of 216 [13.9%] for vaginal delivery vs 55 of 382 [14.4%] for cesarian delivery; *P* = .86]; severe TNH: 5 of 216 [2.3%] for vaginal delivery vs 12 of 382 [3.1%] for cesarian delivery; *P* = .56]) or during the full screening period (72 of 216 [33.3%] for vaginal delivery vs 116 of 382 [30.4%] for cesarian delivery; *P* = .45). Separate analyses for vaginal delivery vs elective or unplanned cesarean delivery are provided in eTable 4 in Supplement 1.

### Exploratory Subgroup Analysis in Neonates With Arterial UCBG of 45 mg/dL or Less

Arterial UCBG level was 45 mg/dL or less in 22 neonates (3.7%) and less than 30 mg/dL in 3 neonates (0.5%). Compared with neonates with levels greater than 45 mg/dL, those with levels of 45 mg/dL or less had significantly higher median venous-arterial UCBG level differences (15 [IQR, 10-23] mg/dL vs 24 [IQR, 17-38] mg/dL) and UCBG extraction rates (18.7% [IQR, 11.9%-25.0%] vs 35.3% [IQR, 27.5%-56.1%]) (*P* < .001 for both) (eTable 5 in Supplement 1).

When comparing neonates with arterial UCBG levels greater than 45 mg/dL with those with levels or 45 mg/dL or less, there were no significant differences in the first blood glucose value (median, 55 [IQR, 46-73] mg/dL vs 63 [IQR, 52-72] mg/dL [*P* = .16]) or in the incidence of TNH (5 of 22 [22.7%] vs 80 of 576 [13.9%] [*P* = .22]) and severe TNH (2 of 22 [9.1%] vs 15 of 586 [2.6%] [*P* = .13]) at the first measurement. The overall incidence of TNH also did not differ significantly between these 2 groups (arterial UCBG level ≤45 mg/dL, 10 of 22 [45.5%] vs >45 mg/dL, 178 of 576 [30.9%] [*P* = .15]). However, severe TNH occurred more frequently in neonates with arterial UCBG levels of 45 mg/dL or less (3 of 22 [13.6%]) vs those with levels greater than 45 mg/dL (19 of 576 [3.3%]) (*P* = .04) (eTable 5 in Supplement 1).

### Estimation of Postnatal Glycemia From UCBG Measurements

AUROC analyses demonstrated that arterial UCBG, venous-arterial difference, and glucose extraction rate were not associated with at least 1 TNH value (≤45 mg/dL or <30 mg/dL; maximum AUROC, 0.64 [95% CI, 0.45-0.81]; *P* = .21) (Table 3). No percentile-based cutoff yielded clinically meaningful sensitivity or positive predictive value in the overall cohort or subgroups (eTables 6-9 in Supplement 1). In a multivariable logistic regression analysis adjusted for gestational age, birth weight standard deviation score, delivery mode, perinatal stress, and maternal diabetes, UCBG parameters were not independently associated with TNH (Table 4).

**Table 3.** ROC and AUROC Analysis for the Discrimination of Transitional Neonatal Hypoglycemia

ROC analysis	Study group						
	Neonates exposed to hypoglycemia risk factors (n = 521)				Neonates unexposed to hypoglycemia risk factors (n = 77)		
	AUROC (95% CI)	*P* value	No. with blood glucose level <30 mg/dL or ≤45 mg/dL	AUROC (95% CI)		*P* value	No. with blood glucose level <30 mg/dL or ≤45 mg/dL
Arterial UCBG level vs first BG level <30 mg/dL	0.39 (0.25-0.53)	.12	17	NC		NA	0
Arterial UCBG level vs first BG level ≤45 mg/dL	0.46 (0.38-0.54)	.30	75	0.47 (0.25-0.69)		.78	10
Arterial UCBG level vs ≥1 BG level <30 mg/dL	0.40 (0.27-0.53)	.14	21	NC		NA	1
Arterial UCBG level vs ≥1 BG level ≤45 mg/dL	0.47 (0.41-0.52)	.21	173	0.41 (0.24-0.58)		.30	15
UCBG extraction rate^a^ vs first BG level <30 mg/dL	0.62 (0.50-0.74)	.06	17	NC		NA	0
UCBG extraction rate^a^ vs first BG level ≤45 mg/dL	0.49 (0.42-0.56)	.86	74	0.61 (0.44-0.77)		.21	10
UCBG extraction rate^a^ vs ≥1 BG level <30 mg/dL	0.57 (0.45-0.70)	.06	19	NC		NA	1
UCBG extraction rate^a^ vs ≥1 BG level ≤45 mg/dL	0.50 (0.45-0.55)	.99	168	0.52 (0.37-0.67)		.82	15
Venous-arterial UCBG level difference vs first BG level <30 mg/dL	0.56 (0.46-0.69)	.22	17	NC		NA	0
Venous-arterial UCBG level difference vs first BG level ≤45 mg/dL	0.47 (0.40-0.54)	.45	74	0.64 (0.46-0.81)		.21	10
Venous-arterial UCBG level difference vs ≥1 BG level <30 mg/dL	0.53 (0.40-0.65)	.67	19	NC		NA	1
Venous-arterial UCBG level difference vs ≥1 BG level ≤45 mg/dL	0.49 (0.44-0.54)	.68	168	0.53 (0.38-0.69)		.69	15

Abbreviations: AUROC, area under the receiver operating characteristics (ROC) curve; BG, blood glucose; NA, not applicable; NC, not computed (due to the small sample size [n ≤ 1]); UCBG, umbilical cord blood glucose.

SI conversion factor: To convert blood glucose to mmol/L, multiply by 0.0555.

^a^ Calculated as [(venous UCBG – arterial UCBG)/venous UCBG] × 100.

**Table 4.** Multivariable Logistic Regression Analysis of UCBG Parameters and Transitional Neonatal Hypoglycemia Adjusted for Risk Factors, Gestational Age, and Mode of Delivery^a^

Parameter	OR (95% CI)	*P* value
Arterial UCBG level vs first BG level <30 mg/dL	0.98 (0.95-1.00)	.11
Arterial UCBG level vs first BG level ≤45 mg/dL	1.00 (0.98-1.00)	.50
Arterial UCBG level vs ≥1 BG level <30 mg/dL	0.98 (0.95-1.00)	.08
Arterial UCBG level vs ≥1 BG level ≤45 mg/dL	1.00 (0.99-1.00)	.58
UCBG extraction rate^b^ vs first BG level <30 mg/dL	1.00 (0.99-1.10)	.07
UCBG extraction rate^b^ vs first BG level ≤45 mg/dL	1.00 (0.99-1.00)	.44
UCBG extraction rate^b^ vs ≥1 BG level <30 mg/dL	1.00 (0.98-1.10)	.40
UCBG extraction rate^b^ vs ≥1 BG level ≤45 mg/dL	1.00 (0.99-1.00)	.27
Venous-arterial UCBG level difference vs first BG level <30 mg/dL	1.00 (0.99-1.10)	.22
Venous-arterial UCBG level difference vs first BG level ≤45 mg/dL	1.00 (0.98-1.00)	.82
Venous-arterial UCBG level difference vs ≥1 BG level <30 mg/dL	1.00 (0.97-1.00)	.78
Venous-arterial UCBG level difference vs ≥1 BG level ≤45 mg/dL	1.00 (0.99-1.00)	.64

Abbreviations: BG, blood glucose; OR, odds ratio; UCBG, umbilical cord blood glucose.

SI conversion factor: To convert blood glucose to mmol/L, multiply by 0.0555.

^a^ Adjustments were made for the following covariates: maternal diabetes, perinatal stress, birth weight standard deviation score, gestational age, and mode of delivery.

^b^ Calculated as [(venous UCBG – Arterial UCBG)/venous UCBG] × 100.

## Discussion

This prospective cohort study is the first, to our knowledge, to examine the association between multiple UCBG parameters and TNH in a large, well-defined population and to provide context-specific percentile-based UCBG reference data. Prior studies^16,17^ were limited by small, heterogeneous cohorts and lacked standardized methodology or reference values.

In utero, fetal plasma glucose level is slightly lower than maternal values.^25^ Glucose-dependent fetal insulin secretion occurs at a lower threshold than postnatally, creating physiologic hyperinsulinemia that regulates fetal growth.^26^

After birth, cessation of placental glucose supply causes a physiologic decline in plasma glucose levels of approximately 20 to 30 mg/dL, reaching a nadir at around 2 hours of life.^4,26^ During this transition, insulin secretion remains elevated, and ketogenesis is suppressed.^4^ These features suggest that TNH reflects persistence of fetal insulin secretion thresholds, hence a continuation of intrauterine physiology into early neonatal life.

UCBG reflects the fetal metabolic state at birth: venous UCBG represents placental glucose delivery, while arterial UCBG reflects blood glucose concentration after fetal glucose extraction. The venous-arterial difference and extraction rate therefore indicate fetal glucose utilization, thus potentially reflecting the fetal insulin secretion at birth. Lower arterial UCBG, greater venous-arterial gradients, and higher extraction rates might therefore discriminate TNH risk.

However, arterial UCBG concentrations, venous-arterial glucose difference, and extraction rate did not estimate TNH with clinically useful sensitivity or specificity in this study. Similarly, Kennedy et al^16^ found limited utility of UCBG extraction rate in discriminating early TNH (AUROC of 0.74 with modest sensitivity and specificity) in a retrospective analysis of 154 newborns. However, their vessel assignment relied solely on pH criteria, excluding samples only when arterial pH was greater than or equal to venous pH.

In contrast, Wang et al^17^ reported moderate predictive performance of arterial UCBG for TNH in a high-risk cohort in China. However, their study’s UCBG levels were considerably higher than ours (85-90 mg/dL vs 67 mg/dL). Although maternal carbohydrate supplementation during labor may contribute, the lack of vessel assignment validation suggests inadvertent inclusion of venous samples.

Venous UCBG primarily reflects maternal rather than fetal metabolism and is therefore less relevant for assessing fetal glucose homeostasis. This may explain the higher UCBG values in the study by Wang et al^17^ and limits the interpretability of their findings. In our study, more than one-quarter of paired samples failed to meet validation criteria, highlighting the risk of vessel misclassification without rigorous quality controls and training. This underscores the practical challenges of using UCBG as a routine screening tool.

Neonates born vaginally had higher venous-arterial UCBG differences and extraction rates than those born by cesarean delivery, likely reflecting metabolic demands and catecholamine surge during labor and birth stress. Despite increased intrauterine glucose utilization, early TNH rates were similar, suggesting effective postnatal adaptation.

Neonates with TNH risk factors had higher UCBG concentrations but similar postnatal glycemia compared with unexposed neonates, resulting in steeper early postnatal glucose decline—yet without differences in UCBG extraction rates. This indicates effective postnatal adaption compensating for increased prenatal glucose exposure.

Although UCBG did not estimate TNH in the overall cohort, arterial UCBG showed a correlation with the initial blood glucose level at 2 to 3 hours of age (ρ = 0.102 [*P* = .01]). Arterial UCBG values of 45 mg/dL or less occurred in only 3.7% (5th percentile, 47 mg/dL). In this subgroup, higher venous-arterial differences and extraction rates were associated with severe TNH, supporting the concept that elevated insulin levels drive greater glucose utilization. However, these findings are exploratory given the small sample size. Very low UCBG values (eg, <5th percentile) may help identify neonates with more pronounced or pathologic forms of hypoglycemia, such as congenital hyperinsulinism, that differ mechanistically from TNH. At-risk neonates (eg, with perinatal stress, small for gestational age) in our cohort had elevated arterial UCBG levels, likely attributable to fetal catecholamine-mediated suppression of intrauterine insulin secretion. In those, TNH may result from hyperresponsive insulin secretion after birth rather than prenatal hyperinsulinemia.^20^ Conversely, congenital hyperinsulinism usually involves already intrauterine insulin secretion, often causing macrosomia through insulin’s growth-promoting effects. UCBG may therefore better indicate postnatal hypoglycemia when pathologic hyperinsulinism is present prenatally. However, this hypothesis requires confirmation in larger prospective studies before clinical application.

### Strengths and Limitations

Strengths of this study include the large prospective cohort providing sufficient power to detect small effects and rigorous validation of arterial and venous cord blood assignment addressing methodologic limitations in previous research.^21,22^ The standardized screening and management protocol for at-risk neonates enhances the generalizability to clinical settings with similar practices. To our knowledge, this study yielded the first percentile-based reference data for arterial and venous UCBG concentration and extraction rates, stratified by risk group and delivery mode, enabling interpretation of UCBG levels in similar clinical and research settings.

This study also has limitations. First, the primary postnatal blood glucose measurement was performed at 2 to 3 hours of life, following current international guidelines that intentionally avoid screening at the physiologic nadir at 1 to 2 hours to minimize overdiagnosis and overtreatment. We therefore assessed associations between cord blood parameters and guideline-prioritized clinical outcomes, and our data might not fully capture the biological glucose nadir at 1 to 2 hours of life. Second, blood glucose measurements were performed using 2 different devices (point-of-care device and blood gas analyzer). While device-related variability cannot be fully excluded, both are validated and widely used in neonatal care and research settings, prioritizing clinical applicability over strict methodologic uniformity. Stratified sensitivity analyses by device were not feasible, as blood glucose levels from blood gas measurements comprised less than 5% of observations and were obtained for clinical indications, introducing systematic bias. Third, the study was conducted at a tertiary center with high rates of cesarean delivery and at-risk births; reference values are therefore specific to this setting. Fourth, exposed neonates underwent more frequent blood glucose measurements than unexposed neonates, reflecting ethical limitations on blood sampling in controls. However, key analyses used umbilical cord blood parameters and first glucose measurement, obtained identically in both groups. Potential bias is therefore limited to analyses of the occurrence of at least 1 hypoglycemic episode. Fifth, the imbalance between exposed and unexposed neonates reflects clinical practice, where screening primarily targets at-risk neonates. A smaller low-risk comparison group was included to allow contextualization of UCBG values in healthy neonates. While this creates unequal group sizes, it reduces precision of between-group comparisons rather than systematic bias. Key analyses focused on within-cohort associations and ROC-based discrimination rather than between-group effects.

## Conclusions

In this prospective cohort study of 598 neonates, UCBG measurements and extraction metrics did not discriminate TNH in at-risk or unexposed neonates born at term and late preterm. These findings demonstrate limitations of single-point assessments for projecting the dynamic, multifactorial postnatal process of TNH. Systematic postnatal glucose monitoring remains the gold standard for identifying and treating at-risk neonates. However, very low UCBG values (eg, <5th percentile) may help distinguish pathologic hypoglycemia (eg, congenital hyperinsulinism) from TNH, warranting validation in larger cohorts with more severe hypoglycemia disorders. Advancing the understanding of neonatal glycemic adaption is critical to optimizing outcomes and minimizing undertreatment and overtreatment of TNH.

## Notes

Supplement 1. **eTable 1.** Comparison of Included vs Excluded Neonates **eTable 2.** Umbilical Cord Blood Parameters With Percentiles for the Total Study Cohort (n = 598) **eTable 3.** Cord Blood Parameters and Glycemia in Different At-Risk Groups Compared With Unexposed Neonates **eTable 4.** Cord Blood Parameters in Neonates According to Delivery Mode **eTable 5.** Exploratory Subgroup Analysis for Study Participants With Arterial UCBG Levels of 45 mg/dL or Less Compared With the Remaining Cohort **eTable 6.** Sensitivity, Specificity, PPV, and NPV of Different Percentile-Based Arterial UCBG Cutoffs for Identifying Neonates With First Blood Glucose Level of 45 mg/dL or Less **eTable 7.** Sensitivity, Specificity, PPV, and NPV of Different Percentile-Based Arterial UCBG Cutoffs for Identifying Neonates With at Least 1 TNH Level of 45 mg/dL or Less **eTable 8.** Sensitivity, Specificity, PPV, and NPV of Different Percentile-Based Glucose Extraction Rate Cutoffs for Identifying Neonates With First Blood Glucose Levels of 45 mg/dL or Less **eTable 9.** Sensitivity, Specificity, PPV, and NPV of Different Percentile-Based Glucose Extraction Rate Cutoffs for Identifying Neonates With at Least 1 TNH Level of 45 mg/dL or Less

Supplement 2.
Data Sharing Statement
