# Supplementary material for: Umbilical Cord Blood Glucose Concentrations and Transitional Neonatal Hypoglycemia
Source: JAMA Netw Open. 2026 Apr 20;9(4):e266170. doi: 10.1001/jamanetworkopen.2026.6170 (PMC13096979; doi:10.1001/jamanetworkopen.2026.6170)
Supplement: Supplement 1. — eTable 1. Comparison of Included vs Excluded Neonates eTable 2. Umbilical Cord Blood Parameters With Percentiles for the Total Study Cohort (n = 598) eTable 3. Cord Blood Parameters and Glycemia in Different At-Risk Groups Compared With Unexposed Neonates eTable 4. Cord Blood Parameters in Neonates According to Delivery Mode eTable 5. Exploratory Subgroup Analysis for Study Participants With Arterial UCBG Levels of 45 mg/dL or Less Compared With the Remaining Cohort eTable 6. Sensitivity, Specificity, PPV, and NPV of Different Percentile-Based Arterial UCBG Cutoffs for Identifying Neonates With First Blood Glucose Level of 45 mg/dL or Less eTable 7. Sensitivity, Specificity, PPV, and NPV of Different Percentile-Based Arterial UCBG Cutoffs for Identifying Neonates With at Least 1 TNH Level of 45 mg/dL or Less eTable 8. Sensitivity, Specificity, PPV, and NPV of Different Percentile-Based Glucose Extraction Rate Cutoffs for Identifying Neonates With First Blood Glucose Levels of 45 mg/dL or Less eTable 9. Sensitivity, Specificity, PPV, and NPV of Different Percentile-Based Glucose Extraction Rate Cutoffs for Identifying Neonates With at Least 1 TNH Level of 45 mg/dL or Less [file jamanetwopen-e266170-s001.pdf]

Supplementary Online Content

Roeper M, Meissner T, Friesl L, et al. Umbilical cord blood glucose concentrations and transitional neonatal hypoglycemia. *JAMA Netw Open.* 2026;9(4):e266170. doi:10.1001/jamanetworkopen.2026.6170

eTable 1. Comparison of Included vs Excluded Neonates

eTable 2. Umbilical Cord Blood Parameters With Percentiles for the Total Study Cohort (n = 598)

eTable 3. Cord Blood Parameters and Glycemia in Different At-Risk Groups Compared With Unexposed Neonates

eTable 4. Cord Blood Parameters in Neonates According to Delivery Mode

eTable 5. Exploratory Subgroup Analysis for Study Participants With Arterial UCBG Levels of 45 mg/dL or Less Compared With the Remaining Cohort

eTable 6. Sensitivity, Specificity, PPV, and NPV of Different Percentile-Based Arterial UCBG Cutoffs for Identifying Neonates With First Blood Glucose Level of 45 mg/dL or Less

eTable 7. Sensitivity, Specificity, PPV, and NPV of Different Percentile-Based Arterial UCBG Cutoffs for Identifying Neonates With at Least 1 TNH Level of 45 mg/dL or Less

eTable 8. Sensitivity, Specificity, PPV, and NPV of Different Percentile-Based Glucose Extraction Rate Cutoffs for Identifying Neonates With First Blood Glucose Levels of 45 mg/dL or Less

eTable 9. Sensitivity, Specificity, PPV, and NPV of Different Percentile-Based Glucose Extraction Rate Cutoffs for Identifying Neonates With at Least 1 TNH Level of 45 mg/dL or Less

This supplementary material has been provided by the authors to give readers additional information about their work.

© 2026 Roeper M et al. *JAMA Network Open*.

eTable 1. Comparison of Included vs Excluded Neonates

	Included (n = 598)	Excluded (n = 420)	
**Characteristics**	**Median (IQR) / N (%)**	**Median (IQR) / N (%)**	***P*-value**
Male sex	332 (55.5%)	216 (51.4%)	.20
Singleton pregnancy	530 (88.6%)	376 (89.5%)	.65
Weeks of gestation [median (IQR)]	39+0 (37+6; 40+0)	39+0 (37+6; 40+0)	.63
Birth weight in gram [median (IQR)]	3300 (2754; 3846)	3213 (2818; 3652)	.19
Birth weight SDS [median (IQR)]	-.13 (-1.1;.93)	-.26 (-.98; .55)	.06
Vaginal Delivery	216 (36.1%)	196 (46.7%)	<.001
Cesarean section	382 (63.9%)	224 (53.3%)	<.001
Elective cesarean section	215 (56.3%)	140 (62.5%)	.16
Unplanned cesarean section	167 (43.7%)	85 (37.9%)	.16
APGAR 1 min [median (range)]	9 (1 – 10)	9 (1 – 10)	.86
APGAR 5 mins [median (range)]	10 (3 – 10)	10 (3 – 10)	.68
APGAR 10 mins [median (range)]	10 (5 – 10)	10 (4 – 10)	.86
Maternal IV glucose infusion during delivery	32 (5.4%)	27 (6.4%)	.47
Transfer to children's hospital	101 (16.9%)	69 (16.4%)	.85
IV Glucose treatment for TNH	44 (7.4%)	42 (10.0%)	.09
Number of risk factors for TNH [median (IQR)]	1 (1;2)	1 (1;2)	.001
Maternal diabetes in pregnancy	228 (38.1%)	137 (32.6%)	.07
Small for gestational age and/or fetal growth restriction	144 (24.1%)	95 (22.6%)	.59
Large for gestational age	108 (18.1%)	46 (11.0%)	.002
Late preterm birth (35+0 - 36+6)	103 (17.2%)	67 (16.0%)	.64
Perinatal Stress	123 (20.6%)	71 (16.9%)	.14
Hypothermia	137 (22.9%)	99 (23.6%)	.81
No risk factors for TNH	77 (12.9%)	84 (20.0%)	.002
Number of BG measurements [median (IQR)]	6 (4-11)	6 (4-11)	.23
Age at first BG measurement [mins] [median (IQR)]	157 (133; 177)	170 (150; 200)	<.001
Value of first BG [mg/dL] [median (IQR)]	63 (52; 72)	61 (50; 71)	.10
Lowest BG value [mg/dL] [median (IQR)]	51 (44; 59)	51 (41; 58)	.41
Age at lowest BG value [mins] [median (IQR)]	379 (176; 869)	496 (188; 1301)	.004
First BG ≤45 mg/dL	85 (14.2%)	74 (17.6%)	.14
First BG <30 mg/dL	17 (2.8%)	12 (2.9%)	.93
≥1 BG ≤45 mg/dL	188 (31.4%)	143 (34.0%)	.38
≥1 BG <30 mg/dL	22 (3.7%)	19 (4.5%)	.50

Abbreviations: BG = Blood glucose, NA = Not applicable, IQR = Interquartile range (25th; 75th percentile), IV = Intravenous, UCBG = Umbilical cord blood glucose, SDS = Standard deviation score. TNH = Transitional neonatal hypoglycemia, SI conversion factors: To convert blood glucose to mmol/L, multiply values by 0.0555. Categorical data are reported as number (percent) and numerical data are reported as median (IQR) unless stated otherwise.

© 2026 Roeper M et al. *JAMA Network Open*.

eTable 2. Umbilical Cord Blood Parameters With Percentiles for the Total Study Cohort (n = 598)

	**Median**	**Percentiles**					
		**5th**	**10th**	**25th**	**75th**	**90th**	**95th**
**Arterial pH**	7.28	7.15	7.18	7.23	7.31	7.33	7.35
**Venous pH**	7.35	7.25	7.28	7.32	7.38	7.40	7.42
**Arterial Base excess [mmol/L]**	-3.3	-10.2	-8.7	-5.7	-1.7	-.5	.2
**Arterial pCO2 [mmHg]**	52.4	41.7	44.4	48.1	57.5	63.0	67.1
**Venous pCO2 [mmHg]**	41.1	30.4	32.9	37.3	44.6	49.2	51.4
**Arterial Lactate [mmol/L]**	3.5	1.6	1.9	2.4	5.0	7.1	7.8
**Venous Lactate [mmol/L]**	3.1	1.4	1.6	2.0	4.5	6.3	7.6
**Arterial UCBG [mg/dL]**	67	47	51	58	84	101	117
**Venous UCBG [mg/dL]**	85	61	65	72	102	119	128
**Δ Venous-Arterial UCBG [mg/dL]**	16	-2	3	10	23	34	39
**Δ Arterial UCBG and first BG [mg/dL]**	6	-25	-19	-7	25	46	59
**UCBG extraction rate [%][a]**	19.0	-1.6	4.0	12.2	25.5	33.3	38.1

Abbreviations: UCBG = Umbilical cord blood glucose, Δ = Difference (Delta).
[a]Calculated as [(Venous UCBG – Arterial UCBG) / Venous UCBG x 100]. SI conversion factors: To convert blood glucose to mmol/L, multiply values by 0.0555.

© 2026 Roeper M et al. *JAMA Network Open*.

eTable 3. Cord Blood Parameters and Glycemia in Different At-Risk Groups Compared With Unexposed Neonates

	Risk factor Maternal Diabetes (n = 228)				Risk factor LGA (n = 108)				Risk factor SGA and/or FGR (n = 144)			
	**Median**	**IQR**	**5th; 95th percentile**	***P* -value[b]**	**Median**	**IQR**	**5th; 95th percentile**	***P* -value[b]**	**Median**	**IQR**	**5th; 95th percentile**	***P* -value[b]**
**Arterial pH**	7.28	7.23; 7.31	7.15; 7.34	.14	7.26	7.22; 7.30	7.14; 7.33	.006	7.28	7.22; 7.31	7.15; 7.35	.20
**Venous pH**	7.35	7.32; 7.38	7.25; 7.42	.006	7.35	7.33; 7.38	7.28; 7.41	.04	7.35	7.31; 7.38	7.23; 7.44	.02
**Arterial Base excess [mmol/L]**	-3.3	-5.7; -1.8	-9.8; -.1	<.001	-3.1	-5.5; -1.7	-10.1; .04	.01	-3.8	-6.6; -2.2	-10.7; -.4	<.001
**Arterial pCO2 [mmHg]**	51.9	48.0; 58.6	40.9; 67.5	.66	56	51.0; 59.9	45.2; 68.4	.004	50.8	47.5; 55.1	41.8; 68.8	.36
**Venous pCO2 [mmHg]**	41.1	37.6; 44.6	31.3; 51.8	.37	42	39.5; 45.5	32.8; 53.5	.04	40.6	36.1; 44.5	27.4; 50.5	.95
**Arterial Lactate [mmol/L]**	3.4	2.5; 4.8	1.6; 7.7	.10	3.1	2.3; 4.6	1.7; 7.0	.46	4.4	3.0; 6.5	1.9; 8.7	<.001
**Arterial UCBG [mg/dL]**	69	59; 89	47; 125	<.001	64	56; 76	44; 103	.32	68	57; 87	46; 118	.01
**Venous UCBG [mg/dL]**	86	75; 106	62; 149	<.001	84	74; 99	62;117	.01	87	68; 104	61; 130	.02
**First BG [mg/dL]**	64	51; 72	33; 90	.79	64	52; 74	41; 85	.50	62	49; 71	31; 91	.56
**Δ Venous-Arterial UCBG [mg/dL]**	15	11; 24	-5; 43	.39	17	13; 27	0; 44	.03	15	9; 22	-4; 38	.94
**Δ Arterial UCBG and first BG [mg/dL]**	7	-6.0; 28	-23; 67	.01	-2	-10; 15	-21; 41	.98	11	-7; 28	-26; 67	.01
**UCBG extraction rate [%][a]**	18.8	12.8; 26.2	-6.0; 38.1	.52	21.8	15.9; 27.6	0; 45.6	.14	17.9	11.4; 25.2	-4.6; 39.8	.27

Abbreviations: BG = Blood glucose, IQR = Interquartile range (25th; 75th percentile), n = Number, UCBG = Umbilical cord blood glucose, Δ = Difference (Delta), LGA = Large for gestational age, SGA = Small for gestational age, FGR = Fetal growth restriction. SI conversion factors: To convert blood glucose to mmol/L, multiply values by 0.0555. [a]Calculated as [(Venous UCBG – Arterial UCBG) / Venous UCBG x 100]. [b]Mann-Whitney test was computed in comparison to the unexposed group. A two-sided *P* <.05 was considered statistically significant. Parameters of the unexposed group are displayed in Table 2.

© 2026 Roeper M et al. *JAMA Network Open*.

eTable 3. Cord Blood Parameters and Glycemia in Different At-Risk Groups Compared With Unexposed Neonates **(continued)**

	Risk factor late preterm (n = 102)				Risk factor perinatal stress (n = 123)			
	**Median**	**IQR**	**5th; 95th percentile**	***P*-value[b]**	**Median**	**IQR**	**5th; 95th percentile**	***P*-value[b]**
**Arterial pH**	7.29	7.25; 7.32	7.14; 7.37	.55	7.26	7.21; 7.30	7.07; 7.35	.002
**Venous pH**	7.36	7.33; 7.38	7.25; 7.43	.07	7.34	7.30; 7.38	7.18; 7.43	<.001
**Arterial Base excess [mmol/L]**	-2.7	-4.7; -1.3	-10.0; .9	.29	-4.1	-7.35; -2.4	-12.5; .3	<.001
**Arterial pCO2 [mmHg]**	52.2	48.7; 56.1	41.0; 63.7	.85	53.3	49.3; 58.6	41.2; 75.1	.20
**Venous pCO2 [mmHg]**	42.1	37.9; 46.1	29.0; 51.3	.13	42.0	37.7; 46.4	28.7; 55.7	.13
**Arterial Lactate [mmol/L]**	2.6	2.0; 3.5	1.5; 6.6	.08	4.5	3.3; 7.1	1.9; 10.2	<.001
**Arterial UCBG [mg/dL]**	66	57; 78	46; 116	.16	77	61; 95	47; 127	<.001
**Venous UCBG [mg/dL]**	79	70; 95	59; 128	.54	92	76; 108	61; 137	<.001
**First BG [mg/dL]**	58	48; 68	29; 80	.05	59	48; 72	29; 97	.40
**Δ Venous-Arterial UCBG [mg/dL]**	13	8; 19	-2; 39	.20	14	7; 21	-2; 41	.35
**Δ Arterial UCBG and first BG [mg/dL]**	9	-4; 24	-25; 57	.02	17	0; 38	-34; 66	<.001
**UCBG extraction rate [%][a]**	16.1	11.6; 23.8	-1.9; 37.5	.04	15.3	7.9; 23.9	-1.7; 35.4	.01

Abbreviations: BG = Blood glucose, IQR = Interquartile range (25th; 75th percentile), n = Number, UCBG = Umbilical cord blood glucose, Δ = Difference (Delta), LGA = Large for gestational age, SGA = Small for gestational age, FGR = Fetal growth restriction. SI conversion factors: To convert blood glucose to mmol/L, multiply values by 0.0555. [a]Calculated as [(Venous UCBG – Arterial UCBG) / Venous UCBG x 100]. [b]Mann-Whitney test was computed in comparison to the unexposed group. A two-sided *P* <.05. Parameters of the unexposed group are displayed in Table 2.

© 2026 Roeper M et al. *JAMA Network Open*.

eTable 4. Cord Blood Parameters in Neonates According to Delivery Mode

	Vaginal Delivery (n = 216)			Elective cesarean delivery (n = 215)			Unplanned cesarean delivery (n = 167)			
	**Median**	**IQR**	**5th; 95th percentile**	**Median**	**IQR**	**5th; 95th percentile**	**Median**	**IQR**	**5th; 95th percentile**	***P*-value[b]**
**Arterial pH**	7.23	7.19; 7.28	7.11; 7.35	7.30	7.27; 7.32	7.22; 7.35	7.29	7.26; 7.32	7.16; 7.35	<.001
**Venous pH**	7.25	7.30; 7.39	7.23; 7.45	7.36	7.34; 7.38	7.28; 7.41	7.35	7.32; 7.37	7.25; 7.40	.001
**Arterial Base excess [mmol/L]**	-6.4	-8.3; -3.9	-11.8; -1.9	-2.0	-3.2; -.9	5.2; .6	-2.9	-4.8; -1.4	-8.9; .8	<.001
**Arterial pCO2 [mmHg]**	53.2	47.3; 59.8	40.0; 70.5	52.1	48.8; 56.3	44.2; 64.7	52.1	47.5; 56.2	41.9; 65.5	.29
**Venous pCO2 [mmHg]**	38.4	34.0; 42.7	27.8; 50.0	42.3	39.7; 45.3	34.1; 53.8	42.0	38.4; 45.8	32.7; 51.0	<.001
**Arterial Lactate [mmol/L]**	5.3	4.2; 7.0	3.2; 9.2	2.5	2.0; 3.1	1.5; 4.5	3.5	2.3; 4.7	1.7; 7.6	<.001
**Arterial UCBG [mg/dL]**	82	67; 96	54; 135	58	51; 63	44; 74	74	63; 87	49; 114	<.001
**Venous UCBG [mg/dL]**	102	89; 117	74; 151	71	65; 78	59; 95	89	76; 101	65; 123	<.001
**First BG [mg/dL]**	66	54; 76	38; 93	61	50; 70	32; 81	62	52; 73	35; 93	.005
**Δ Venous-Arterial UCBG [mg/dL]**	22	11; 32	-7; 47	14	10; 19	3; 29	14	9; 19	-1; 34	<.001
**Δ Arterial UCBG and first BG [mg/dL]**	16	0; 39	-20; 73	-3	-12; 7	-31; 32	14	-4; 29	-26; 58	<.001
**UCBG extraction rate [%][a]**	22.9	9.1; 30.4	-7.8; 42.3	20.0	14.9; 24.7	4.8; 35.1	16.0	10.3; 21.6	-.8; 32.7	<.001

Abbreviations: BG = Blood glucose, IQR = Interquartile range (25th; 75th percentile), n = Number, UCBG = Umbilical cord blood glucose, Δ = Difference (Delta).
SI conversion factors: To convert blood glucose to mmol/L, multiply values by 0.0555. [a]Calculated as [(Venous UCBG – Arterial UCBG) / Venous UCBG x 100]. [b]Kruskal Wallis test was computed for comparison of the three delivery modes. A two-sided *P* <.05 was considered statistically significant.

© 2026 Roeper M et al. *JAMA Network Open*.

eTable 5. Exploratory Subgroup Analysis for Study Participants With Arterial UCBG Levels of 45 mg/dL or Less Compared With the Remaining Cohort

	Arterial UCBG ≤45 mg/dL (n = 22)		Arterial UCBG >45 mg/dL (n = 576)		
**Characteristics**	**Median (IQR) / N (%)**	**95% CI[c]**	**Median (IQR) / N (%)**	**95% CI[c]**	***P*-value[b]**
**Unexposed group**	2 (2.6%)	NA	75 (97%)	NA	.59
**Exposed group**	20 (3.8%)	NA	501 (96.2%)	NA	.59
**Age at first BG measurement [mins]**	141 (131; 164)	119 – 163	157 (133; 177)	148 – 155	.17
**Value of first BG [mg/dL]**	55 (46; 73)	48 – 65	63 (52; 72)	61 – 64	.16
**Lowest BG value [mg/dL]**	47 (41; 56)	40 – 53	51 (44; 59)	50 – 52	.14
**First BG ≤ 45 mg/dL**	5 (22.7%)	9.2 – 42.9	80 (13.9%)	11.2 – 16.9	.22
**First BG <30 mg/dL**	2 (9.1%)	1.9 – 26.1	15 (2.6%)	1.5 – 4.2	.13
**≥1 BG ≤45 mg/dL**	10 (45.5%)	26.3 – 65.7	178 (30.9%)	27.2 – 34.8	.15
**≥1 BG <30 mg/dL**	3 (13.6%)	4.0 – 32.1	19 (3.3%)	2.1 – 5.0	.04
**Δ Venous-Arterial UCBG [mg/dL]**	24 (17; 38)	21 – 36	15 (10; 23)	15 – 17	<.001
**Δ Arterial UCBG and first BG [mg/dL]**	-15 (-36; -5)	-27 – -7	7 (-7; 25)	9 – 13	<.001
**UCBG extraction rate [%][a]**	35.3 (27.5; 56.1)	32.3 – 47.1	18.7 (11.9; 25.0)	16.5 – 18.8	<.001

Abbreviations: BG = Blood glucose, IQR = Interquartile range (25th; 75th percentile), n = Number, NA = not applicable, UCBG = Umbilical cord blood glucose, Δ = Difference (Delta), CI = Confidence interval.

SI conversion factors: To convert blood glucose to mmol/L, multiply values by 0.0555. [a]Calculated as [(Venous UCBG – Arterial UCBG) / Venous UCBG x 100]. Categorical data are reported as number (percent) and numerical data are reported as median (IQR). [b]Mann-Whitney test and CHI-Squared or Fisher’s Exact Tests. [c]Clopper-Pearson exact method (binominal). A two-sided *P* <.05 was considered statistically significant.

© 2026 Roeper M et al. *JAMA Network Open*.

eTable 6. Sensitivity, Specificity, PPV, and NPV of Different Percentile-Based Arterial UCBG Cutoffs for Identifying Neonates With First Blood Glucose Level of 45 mg/dL or Less

	Total cohort (n = 598)				Exposed (n = 521)				Unexposed (n = 77)			
**Art. UCBG**	**Sensitivity**	**Specificity**	**PPV**	**NPV**	**Sensitivity**	**Specificity**	**PPV**	**NPV**	**Sensitivity**	**Specificity**	**PPV**	**NPV**
<47 mg/dL (<5th Percentile)	8.2	95.1	21.9	86.2	6.7	95.3	19.2	86.0	20	94	33.3	88.7
<51 mg/dL (<10th Percentile)	20.0	90.1	25.0	87.2	18.7	90.8	25.5	86.9	30	85.1	23.1	89.1
<58 mg/dL (<25th Percentile)	35.3	73.3	18.0	87.2	33.3	75.1	18.4	87.0	50	61.1	16.1	89.1
<67 mg/dL (<50th Percentile)	52.9	49.3	14.8	86.3	53.3	52.2	15.8	86.9	50	29.9	9.6	80
<84 mg/dL (<75th Percentile)	72.9	24.4	13.8	84.5	70.6	26.2	13.9	84.2	90	11.9	13.2	88.9
<101 mg/dL (<90th Percentile	91.8	10.1	14.5	88.1	90.7	11.0	14.6	87.5	100	4.5	13.5	100
<117 mg/dL (<95th Percentile)	92.9	3.9	13.8	76.9	92.0	4.5	13.9	76.9	100	NA	13	NA

Abbreviations: PPV = Positive predictive value, NPV = Negative predictive value. NA = Not available. All results are percent. SI conversion factors: To convert blood glucose to mmol/L, multiply values by 0.0555.

© 2026 Roeper M et al. *JAMA Network Open*.

eTable 7. Sensitivity, Specificity, PPV, and NPV of Different Percentile-Based Arterial UCBG Cutoffs for Identifying Neonates With at Least 1 TNH Level of 45 mg/dL or Less

	Total cohort (n = 598)				Exposed (n = 521)				Unexposed (n = 77)			
**Art. UCBG**	**Sensitivity**	**Specificity**	**PPV**	**NPV**	**Sensitivity**	**Specificity**	**PPV**	**NPV**	**Sensitivity**	**Specificity**	**PPV**	**NPV**
<47 mg/dL (<5th Percentile)	6.9	95.4	40.6	69.1	5.8	95.4	38.5	67.1	20	95.2	50	83.1
<51 mg/dL (<10th Percentile)	15.4	90.5	42.6	70	14.5	91.4	45.5	68.2	26.7	85.5	30.8	82.8
<58 mg/dL (<25th Percentile)	34.6	75.1	38.9	71.5	32.9	77.3	41.9	69.9	53.3	62.9	25.8	84.8
<67 mg/dL (<50th Percentile)	54.8	50.4	33.4	96.1	53.8	54	36.8	70.1	66.7	32.3	19.2	80
<84 mg/dL (<75th Percentile)	72.9	23.3	30.4	15.7	71.1	25.6	32.2	64	93.3	12.9	20.6	88.9
<101 mg/dL (<90th Percentile	89.9	9.8	31.4	67.8	89	10.6	33.1	66.1	100	4.8	20.3	100
<117 mg/dL (<95th Percentile)	92.6	2.9	30.4	46.2	91.9	3.4	32.1	46.2	100	NA	19.5	NA

Abbreviations: PPV = Positive predictive value, NPV = Negative predictive value. NA = Not available. TNH = Transitional neonatal hypoglycemia. All results are percent. SI conversion factors: To convert blood glucose to mmol/L, multiply values by 0.0555.

© 2026 Roeper M et al. *JAMA Network Open*.

eTable 8. Sensitivity, Specificity, PPV, and NPV of Different Percentile-Based Glucose Extraction Rate Cutoffs for Identifying Neonates With First Blood Glucose Levels of 45 mg/dL or Less

	Total cohort (n = 598)				Exposed (n = 521)				Unexposed (n = 77)			
**Glucose Extraction rate in %**	**Sensitivity**	**Specificity**	**PPV**	**NPV**	**Sensitivity**	**Specificity**	**PPV**	**NPV**	**Sensitivity**	**Specificity**	**PPV**	**NPV**
>4 (10th Percentile)	92.9	9.9	14.6	89.5	92	10.1	14.7	88.2	100	9	13	100
>12 (25th Percentile)	80	24	14.8	87.9	77.3	24	14.6	86.3	100	23.9	16.4	100
>19 (50th Percentile)	47.9	48	13.6	85.1	46.7	48.7	14.4	84.4	70	43.3	15.6	90.6
>25 (75th Percentile)	28.2	71	13.9	85.6	28	69.7	13.5	85.2	30	79.1	17.6	88.3
>33 (90th Percentile)	8.2	85.6	8.6	84.9	8	84.1	7.8	84.5	10	95.5	25	87.7
>38 (95th Percentile)	7.1	91.2	11.8	85.6	6.7	90.1	10.2	85.2	10	98.5	50	88

Abbreviations: PPV = Positive predictive value, NPV = Negative predictive value. All results are percent. SI conversion factors: To convert blood glucose to mmol/L, multiply values by 0.0555.

© 2026 Roeper M et al. *JAMA Network Open*.

eTable 9. Sensitivity, Specificity, PPV, and NPV of Different Percentile-Based Glucose Extraction Rate Cutoffs for Identifying Neonates With at Least 1 TNH Level of 45 mg/dL or Less

	Total cohort (n = 598)				Exposed (n = 521)				Unexposed (n = 77)			
**Glucose Extraction rate in %**	**Sensitivity**	**Specificity**	**PPV**	**NPV**	**Sensitivity**	**Specificity**	**PPV**	**NPV**	**Sensitivity**	**Specificity**	**PPV**	**NPV**
>4 (10th Percentile)	92	10.2	32	73.3	91.3	10.3	33.6	70.6	100	9.7	21.1	100
>12 (25th Percentile)	77.1	23.7	31.7	69.3	76.3	23.9	33.2	66.9	86.7	22.6	19.5	87.5
>19 (50th Percentile)	50	47.6	30.4	67.5	49.1	48.6	32.2	65.8	60	41.9	20	81.3
>25 (75th Percentile)	29.3	68.7	29.3	68.7	30.1	70.1	33.3	66.8	20	77.4	17.6	80
>33 (90th Percentile)	12.3	68.1	12.2	68.1	12.7	84.2	28.6	66	6.7	95.2	25	80.8
>38 (95th Percentile)	9	91.7	33.3	68.7	9.2	90.5	32.7	66.7	6.7	98.4	50	81.3

Abbreviations: PPV = Positive predictive value, NPV = Negative predictive value, TNH = Transitional neonatal hypoglycemia. All results are percent. SI conversion factors: To convert blood glucose to mmol/L, multiply values by 0.0555.

© 2026 Roeper M et al. *JAMA Network Open*.
