# Supplementary material for: Umbilical Cord Blood Glucose Concentrations and Transitional Neonatal Hypoglycemia
Source: JAMA Netw Open. 2026 Apr 20;9(4):e266170. doi: 10.1001/jamanetworkopen.2026.6170 (PMC13096979; doi:10.1001/jamanetworkopen.2026.6170)
Supplement: Supplement 2. — Data Sharing Statement [file jamanetwopen-e266170-s002.pdf]

Data Sharing Statement

Roeper. Umbilical Cord Blood Glucose Concentrations and Transitional Neonatal Hypoglycemia. *JAMA Netw Open*. Published April 20, 2026.
doi:10.1001/jamanetworkopen.2026.6170

Data
Data available: Yes
Data types: Deidentified participant data
How to access data: Deidentified individual participant data (including data dictionaries) will be made available upon publication to researchers who provide a methodologically sound proposal for use in achieving the goals of the approved proposal. Proposals should be submitted to marcia.roeper@med.uni-duesseldorf.de
When available: With publication

Supporting Documents
Document types: None

Additional Information
Who can access the data: researchers whose proposed use of the data has been approved
Types of analyses: for a methodologically sound proposal that has been approved by the authors.
Mechanisms of data availability: after approval of a proposal and with signed data access agreement.
